# Efficacy of Subcutaneous Closed Suction Drain in Reduction of Postoperative Surgical Site Infection

**DOI:** 10.1055/s-0041-1735900

**Published:** 2021-10-12

**Authors:** R. Harish, Farah Naaz Kazi, J.V. Pranav Sharma

**Affiliations:** 1 Department of General Surgery, Vydehi Institute of Medical Sciences and Research Centre, Bangalore, Karnataka, India

**Keywords:** SSI, subcutaneous suction drain, exploratory laparotomy, microorganism, co-morbidity

## Abstract

**Background** Surgical site infections (SSIs) are the infections of wound after an invasive operative approach. It remains to be a major morbidity for patients undergoing surgeries although there have been tremendous improvements in the surgical techniques.

Different interventions to suppress the selective serotonin reuptake inhibitors have been proposed. Many of them have been routinely used by surgeons like minimizing shaving, hand washing, and preoperative antibiotics and these are well accepted. Drains are used in major abdominal surgeries, hernia repairs, breast surgeries reducing collections in closed areas. 1 Hematoma, serous fluid, and dead space in surgical incision wounds raise the risk of infection as they serve as the platform for microbial growth. Studies have proved that the usage of subcutaneous drains has lowered the chances of infection.

**Results** The patients in the case group had lower incidence of SSI compared with the control group. The patients in the case group had subcutaneous drain which drained any collection that developed in the subcutaneous space. When the incidence of SSI was compared between the emergency cases and elective cases, the emergency cases showed higher propensity for SSI and increased rate for patients who had co-morbidities like diabetes mellitus, hypertension, etc. The most common organism isolated from the SSI was found to be *Escherichia coli* . It was also noted that the mean number of days of hospital stay was comparatively higher for the patients who developed SSI compared with patients who did not develop SSI.

**Conclusion** Thus the presence of SSI adds morbidity to the patient and the patients who undergo major surgeries are likely to develop SSI postoperatively. The presence of subcutaneous closed suction drain helps in reducing the SSI to a certain extent.

## Introduction

The main concern in postoperative patients remains the wound healing and its complications as it tends to increase the morbidity in patients. Wound management forms the pillar of surgical practice and wound-related infections and their management still remain to be a grueling task. 1

One such major postoperative complication is surgical site infections (SSIs). SSIs are the infections in which the microorganisms invade the tissues within 30 days where the surgery has taken place involving the superficial layers and 30 or 90 days for the deep layers. 2 SSIs are further divided into two types: incisional and organ/space. While incisional SSIs are restricted to surgical sites, they can be subcategorized into superficial and deep SSIs. The superficial SSIs affect the skin and superficial fascia; the deep SSIs involve the infection of fascial and muscular layer. Organ/space SSIs infect any tissue below the fascial layer which is involved in the surgical procedure within 30 or 90 days after the surgery. 2 The worldwide incidence of SSI ranges between 0.5 and 15%, while in India, it shows a significant increase of 23 to 38%. 1 3 The potential for SSI development following surgery depends upon the virulence of the microorganisms and their inoculums size. 4 The risk of infection rises due to increased dead space, hematoma or devitalized tissue, all consequences of inadequate surgical procedures. It holds good for any foreign material like drains or sutures as well. It has also been noted that patients with high BMI, known history of alcoholism, chronic heart disease, and diabetes form major contributing factors to the development of SSI. 5 6 This is mainly because they cause a widespread decrease in the immune function thereby causing delayed wound healing. The type of wound and surgeries also plays an important role; a contaminated wound undergoing an emergency surgery (for example, emergency abdominal laparotomy) is more likely to develop SSI after the surgery as compared with an elective surgery on a clean injury (hysterectomy). This is due the presence of microorganisms present in contaminated wounds which may have entered the blood stream causing SSI. These infections present in the form of pain, erythema, fever, pus, or discharge from wound, dehiscence.

To reduce these, several approaches have been employed like adequate hand hygiene, hair removal, and chlorhexidine wash with antibiotic cover before the surgery. 1 7 However, the placement of subcutaneous drain in surgical wounds after the surgery has seemed to be quite promising especially in emergency laparotomies. It is based on the principle of removing the collected fluid or debris and closing the dead space in the subcutaneous plane which in turn will reduce the possibilities of infection and wound complications. 8 The drain output is then monitored appropriately.

**Fig. 1:**
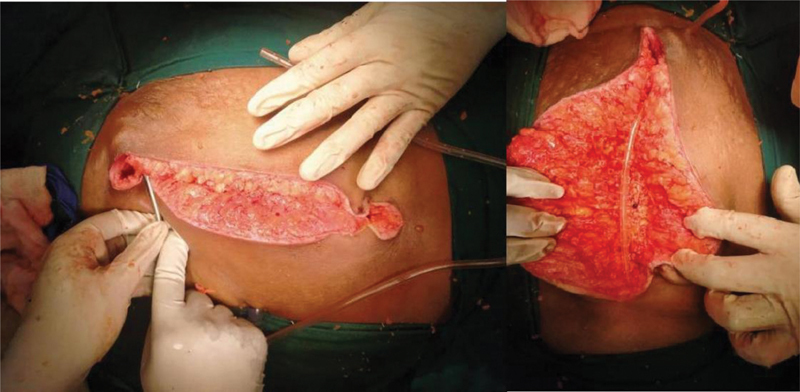
Placement of subcutaneous suction drain.

These drains under flaps can be one of the ways to tackle seroma. It can then be removed under sterile conditions and placement of a pressure dressing. If the seroma gets collected again, then it should be removed by opening of the incision. If seroma reaccumulates after two aspirations, then it should be evacuated by opening the incision and packing the wound with saline gauze so that secondary healing is possible . Hematoma can be prevented by correcting the problems associated with the clotting factors. 9 The safest option for high-risk patients is interrupted sutures or synthetic mesh. This along with moist surgical gauze, iodophor dressing, and a continuous suctioning has proved to be extremely beneficial. The wound can be closed in 7 to 10 days. However, if the wound cannot be closed, then it is allowed to undergo granulation and then closed by skin graft. Some studies have shown that the use of subcutaneous catheter with antibiotic cover and saline irrigation is only effective in case of dirty wounds 10 ( Fig. 1 ).

## Aims and Objectives of the Study

The objectives of the study are given below:

1. To assess the efficacy of subcutaneous wound drainage in the reduction of SSIs.
2. To assess the role of predictive factors in wound infection like diabetes.
3. To compare the rate of SSI between elective and emergency explorative laparotomy surgeries.
4. To identify the most common organism in SSIs.

## Methodology

This is a case–control study of 100 patients undergoing midline exploratory laparotomy surgeries admitted in the surgical department of VIMS and RC between November 2018 and July 2020. All admitted patients underwent clinical examination with relevant investigations after obtaining an informed written consent. Among these, 50 patients were randomly selected and closed subcutaneous suction drain placed before the skin closure, while for the other 50 patients subcutaneous drain was not placed. The patients for whom subcutaneous drain was placed were considered as cases while the other group of patients was considered as control group. The drains in the cases were kept for a duration of 7 to 15 days (average 5.2 days).

## Study Design

Type of study: Prospective case–control study with simple randomization.
Cases: Patients with subcutaneous suction drains.
Controls: Patients without subcutaneous suction drains.

### Inclusion Criteria

1. Age group above 18 years.
2. Patients undergoing emergency and elective laparotomy surgeries.

### Exclusion Criteria

1. Patients less than 18 years.
2. Patients above 80 years.
3. Patients with immunocompromised status like HIV, radiotherapy, or chemotherapy.
4. Re-done laparotomy surgeries.

## Results and Observations

### Statistical Analysis 11 12 13

Data was entered into Microsoft excel data sheet and was analyzed using SPSS 22 version software. Categorical data was represented in the form of frequencies and proportions. *Chi-square test* was used as test of significance for qualitative data. Continuous data was represented as mean and standard deviation. *Independent t-test or Mann-Whitney U test* was used as test of significance to identify the mean difference between two quantitative variables and qualitative variables, respectively.

### Graphical Representation of Data

MS Excel and MS word were used to achieve various types of graphs and ROC curve *p* -value of <0.05 was considered as statistically significant after assuming all the rules of statistical tests.

### Statistical Software

MS Excel, SPSS version 22 **(** IBM SPSS Statistics, Somers NY) was used to analyze data.

There were 26 patients under 40 years and 24 patients above 40 years in the cases group while there were 28 patients below 40 years and 22 patients in the control group ( Table 1 ). There was no significant difference in the age distribution between the two groups in the study.

**Table 1:** Age distribution between two groups

		Group			
		Cases		Controls	
		Count	%	Count	%
Age	≤30 y	15	30%	12	24%
	31–40 y	11	22%	16	32%
	41–50 y	10	20%	14	28%
	51–60 y	8	16%	6	12%
	>50 y	6	12%	2	4.%

Note: *χ* ^2^ = 4.212, d *f* = 4, *p* = 0.378.

There was no significant difference in age group distribution between the two groups. The mean age in the cases was 40.94 ± 15.10 while the mean age in the control group was 39.54 ± 10.54 ( Table 2 ). There was no significant difference in mean age comparison between the two groups.

**Table 2:** Mean age distribution between two groups

	Group				*p* -Value
	Cases		Controls		
	Mean	SD	Mean	SD	
Age	40.94	15.10	39.54	10.54	0.592

The mean duration of stay of the patients in the case group was 8.84 ± 2.85 days and mean duration of stay of the patients in the control group was 11.2 ± 4.85 days ( Table 3 ). Here it was observed that the duration of stay in the control group was around 12 days. On an average, the patients in the case group had 9 days and thus the patients in the control group had a longer stay compared with case group. The patients without a subcutaneous suction drain had developed SSI comparatively more than patients with a subcutaneous suction drain. The presence of SSI led to more morbidity of the patient which led to greater number of days of hospital stay. There was a significant difference in mean duration of stay comparison between the two groups.

**Table 3:** Mean duration of stay comparison between the two groups

	Group				*p* -Value
	Cases		Controls		
	Mean	SD	Mean	SD	
Duration of stay	8.84	2.85	11.20	4.85	0.004*

Note: *p* -Value is below 0.05.

In the case group, there were eight patients who were hypertensive. Thirteen patients had diabetes mellitus alone. In the control group, eight patients had hypertension and 12 patients had diabetes mellitus. There was no significant difference in the co-morbidity distribution among both the groups ( Table 4 ). Also, the two groups of patients in the cases did not show any major difference in the presence of SSI. Out of these, two out of the eight patients with hypertension had SSI while four out of the 13 amongst the diabetics showed SSI. This is in stark contrary to Flynn who stated that SSIs were more common in diabetic patients as compared with nondiabetics. 14 However, some authors showed no disparity of co-morbidities in the development of SSI. 15 There was no significant difference in co-morbidities distribution between the two groups. The mean collection found in the drains in the cases group was 42.54 ± 12.59 mL. It was also found that there was more collection in the patients with co-morbidities like diabetics or hypertension (55.42 ± 2 mL) as compared with nondiabetics and nonhypertensives (39.4 ± 23 mL) and similarly more collection in the patients who had undergone emergency exploratory laparotomy (56.23 ± 3 mL) in comparison to those who underwent elective surgeries (37 ± 4 mL).

**Table 4:** Co Morbidities distribution between the two groups

		Group				Chi-square
		Cases		Controls		
		Count	%	Count	%	
Hypertensive	No	42	84%	42	84%	χ2 = 0.000, df = 1, *p* = 1.00.
	Yes	8	16%	8	16%	
DM	No	37	74%	38	76%	χ2 = 0.053, df = 1, *p* = 0.817.
	Yes	13	26%	12	24%	

Abbreviation: DB, diabetes mellitus.

The collection was serosanguinous in all the cases which gradually decreased by postoperative day 5.

Among the case group, 22 patients underwent elective surgery while 28 patients underwent emergency surgery. In the control group, 25 patients underwent elective surgery, and 25 patients underwent emergency surgery. In the cases group, there was more collection in the drain in patients who underwent emergency surgery (56.23 ± 3 mL) compared with patients who underwent elective surgery (36.8 ± 4 mL) ( Table 5 ).

**Table 5:** Type of surgery distribution between the two groups

		Group			
		Cases		Controls	
		Count	%	Count	%
Type of surgery	Elective	22	44%	25	50%
	Emergency	28	56%	25	50%

Note: *χ* ^2^ = 0.361, df = 1, *p* = 0.548.

There was no significant difference in the type of surgery distribution between the two groups.

In the cases group, seven patients (14%) developed SSI while 43 (86%) patients did not develop any. Among the control group, 21 patients (42%) developed SSI while 29 patients (58%) did not develop any infection. There was a significant difference in the SSI distribution between the two groups. Thus, the control group had more patients developing SSI compared with the patients in the case group for whom subcutaneous closed suction drain was put. Similarly in the study done by Ishikawa et al, 14.7% of the patients developed incisional SSI among all the colorectal surgeries they have included which incorporated drains in all their patients. 16 In a study by Fujii et al with high-risk patients, there was a decrease in the number of patients with SSI in the drain group 17 ( Table 6 ).

**Table 6:** SSI distribution between the two groups

		Group			
		Cases		Controls	
		Count	%	Count	%
SSI	Absent	43	86%	29	58%
	Present	7	14%	21	42%

Abbreviation: SSI, surgical site infection.

Note: *χ* ^2^ = 9.722, df = 1, *p* = 0.002*.

In the case group, seven patients had SSI, out of whom *Escherichia coli* was isolated in five patients and *Staphylococcus aureus* was isolated in two patients. In the control group, 21 patients developed SSI in whom nine patients (18%) had *E. coli* , four patients (8%) had *Enterococcus faecalis* , four patients (8%) had *S. aureus* , three patients (6%) had *Pseudomonas aeruginosa* , and one patient (2%) had *Acinetobacter baumannii* . The most common organism isolated in the study was *E. coli* and next common organism was *S. aureus* ( Table 7 ). In the study done by Ishikawa et al, the organisms isolated were *Enterococcus spp* . (71.4%), *S. aureus* , *Staphylococcus epidermidis* (7.1%), and *P. aeruginosa* (7.1%). 16

**Table 7:** Organisms Isolated between the two groups

		Group			
		Cases		Controls	
		Count	%	Count	%
Organism isolated		43	86%	29	58%
	*Acinetobacter boumani*	0	0%	1	2%
	*Enterococcus faecalis*	0	0%	4	8%
	*Escherichia coli*	5	10%	9	18%
	*Pseudomonas aeruginosa*	0	0%	3	6%
	*Staphylococcus aureus*	2	4%	4	8%

Note: *χ* ^2^ = 12.532, d *f* = 5, *p* = 0.028*.

There was a significant difference in the organisms isolated between the two groups.

The presence of SSIs with respect to the type of surgery was also observed. Out all the elective cases, seven patients (14.89%) had SSI while 40 patients did not have any. Out of the 53 patients who underwent emergency surgery, 21 patients (39.62%) developed SSIs while 32 patients did not have any ( Table 8 ). As per the results, emergency surgeries had higher incidence of SSIs compared with elective surgeries. This was due to greater number of contaminated cases like perforation, blunt trauma abdomen compared with clean cases in the elective list like mesenteric cyst excision, gastric outlet obstruction, etc. 5 6 18 19 There was a significant difference in SSI distribution with respect to the type of surgery.

**Table 8:** SSI distribution with respect to type of surgery

		Type of surgery			
		Elective		Emergency	
		Count	%	Count	%
SSI	Absent	40	85%	32	60%
	Present	7	15%	21	40%

Abbreviation: SSI, surgical site infection.

Note: *χ* ^2^ = 7.556, d *f* = 1, *p* = 0.006.*

The observations were made regarding SSIs with respect to duration of stay. In this particular study, the patients who developed SSI had a mean duration of stay of 15.36 ± 3.27 days and the patients who did not develop surgical site infection had a mean duration of 7.94 ± 2.03 days. Thus, the patients who developed SSI had a longer duration of hospital stay compared with patients who did not develop SSI ( Table 9 ).

**Table 9:** SSI distribution with respect to duration of stay

		Duration of stay		*p* -Value
		Mean	SD	
SSI	Absent	7.94	2.03	<0.001*
	Present	15.36	3.27	

Abbreviation: SSI, surgical site infection.

Observations regarding the type of wounds were also made between the elective and emergency surgeries which showed that the dirty wounds in both the surgeries were high similar to the studies made by Farnell. 10 Out of the total 100 patients, half of the patients were placed on drains. It was observed that amongst the seven patients who developed SSI after an elective surgery ( Tables 6 and 8 ), five were dirty wounded and two were contaminated wounds with no drains. While out of the 21 patients who underwent emergency surgery and developed SSI ( Tables 6 and 8 ), one was clean contaminated, eight were contaminated, and 12 were dirty wounds without drains ( Table 10 ).

**Table 10:** Type of wound between the elective and emergency surgeries

		Surgery			
		Elective		Emergency	
		Count	%	Count	%
Type of wound	Clean	8	16%	3	6%
	Clean—contaminated	6	12%	5	10%
	contaminated	10	20%	19	38%
	Dirty	26	52%	23	46%

Note: *χ* ^2^ = 5.3404, d *f* = 4, *p* = 0.148.*

## Discussion

SSI is one of the leading causes of morbidity in emergency laparotomy. An extensive amount of research has been done in terms of its epidemiology, prevention, and treatment for the SSIs. Amongst the various surgeries, colorectal surgeries have shown to have a high incidence of SSI due to the organisms residing in the intestines. 20 21 In an attempt to find out the risk factors causing SSI, Cruse and Foord showed that the average infection rate was 4.8% in the various surgical fields, with increasing infections in elderly, long duration of hospital stay, and operations. 22 However, their findings showed that the rate of infection also increased with the usage of drains which differ from our results.

In our research we found that SSI rates came up to 14% in patients with a subcutaneous drain and 42% in those without a drain. Also, in our study, there were 26 patients under 40 years and 24 patients above 40 years in the cases group while there were 28 patients below 40 years and 22 patients in the control group ( Table 1 ). There was no significant difference in the age distribution between the two groups in the study. The mean age in the cases was 40.94 ± 15.10 while the mean age in the control group was 39.54 ± 10.54 ( Table 2 ). There was no significant difference in mean age comparison between the two groups.

SSI was also shown to be prevalent in obese patients in studies shown by Sugiura et al. 23 24 25 This is consistent with our analysis as well. However, hypertensive and diabetic patients did not seem to have been significant factors for the development of SSI. Previous studies have also revealed that SSI development leads to longer hospital stay. In the present study we found out that this was true. The patients who developed SSI had a mean duration of stay of 15.36 ± 3.27 days and the patients who did not develop surgical site infection had a mean duration of 7.94 ± 2.03 days. This is mainly due to the fact that SSI management involves close monitoring, repeated dressings, and antibiotic treatment.

Suragul and his colleagues have stated that the cause of SSI was polymicrobial with 48% positive cultures. The most common organisms being *Enterococcus* , *E. coli* , and *Klebsiella pneumonia* which are the normal inhabitants of the intestines causing SSI in abdominal surgeries. 26 While it has been pseudomonad and serratia in thyroid surgeries 27 and *S. aureus* in orthopedic surgeries, 28 our study has shown that the most common organism isolated in the study was *E. coli* and next common organism was *S. aureus* ( Table 7 ).

There have been disputing results in terms of the association of SSI and subcutaneous drains in the past where Fujii et al 17 found a drastic reduction in SSI with subcutaneous drains, Baier did not agree with it. 29 Pan did his research on the patients with ileostomy reversal and agreed with Fujii et al. 30 He found his patients without a drain to develop SSI at the rate of 12.5% while those with a drain at 1.2%. We found similar results in our study.

## Conclusion

Subcutaneous suction drains have proved to reduce SSI in a large number of patients. In our study, the co-morbid patients, emergency cases, and colorectal surgeries had a higher incidence of developing SSI. The most common organism isolated was *E. coli* followed by *S. aureus* . The assessment of these variables therefore helps in providing a prophylactic treatment to reduce the mortality in the high-risk patients.
